# Evaluating automatic sentence alignment approaches on English-Slovak sentences

**DOI:** 10.1038/s41598-023-47479-w

**Published:** 2023-11-17

**Authors:** Frantisek Forgac, Dasa Munkova, Michal Munk, Livia Kelebercova

**Affiliations:** 1 Faculty of Natural Sciences and Informatics, Constantine the Philosopher University in Nitra, Nitra, Slovakia; 2 Science and Research Centre, University of Pardubice, Pardubice, Czech Republic

**Keywords:** Mathematics and computing, Computer science, Scientific data, Software, Statistics

## Abstract

Parallel texts represent a very valuable resource in many applications of natural language processing. The fundamental step in creating parallel corpus is the alignment. Sentence alignment is the issue of finding correspondence between source sentences and their equivalent translations in the target text. A number of automatic sentence alignment approaches were proposed including neural networks, which can be divided into length-based, lexicon-based, and translation-based. In our study, we used five different aligners, namely Bilingual sentence aligner (BSA), Hunalign, Bleualign, Vecalign, and Bertalign. We evaluated both, the performance of the Bertalign in terms of accuracy against the up to now employed aligners as well as among each other in the language pair English-Sovak. We created our custom corpus consisting of texts collected in 2021 and 2022. Vecalign and Bertalign performed statistically significantly best and BSA the worst. Hunalign and Bleualign achieved the same performance in terms of F1 score. However, Bleualign achieved the most diverse results in terms of performance.

## Introduction

In both analytical and inflectional languages, aligning parallel texts involves a systematic, multi-level approach. This alignment procedure initiates at a broader scale, matching entire documents. It then progresses to a more detailed focus, aligning individual paragraphs within those matched documents. As the process continues, it becomes finer, pairing corresponding sentences and ultimately aligning individual words. The process starts at the general level of alignment and increases in granularity of alignment at subsequent level, i.e., from document alignment through paragraph to sentence and word alignments^1^.

Sentence alignment is the task of taking parallel documents, which have been split into sentences, and finding a bipartite graph which matches minimal groups of sentences that are translations of each other^2^. It consists of finding correspondences (alignments) between logical units, i.e., between mutual translations of source and target texts^3^. The most common alignment is 1 to 1 alignment, but there exists a significant presence of complex alignment, such as 1 to 0 or vice versa 0 to 1 and also many to many alignment, depending on the source, target languages (genre and text type) and translator competence^4^. For instance, Sennrich and Volk^5^ manually aligned a set of 1000 sentences and found that only 74% of sentence alignments were 1 to1. Depending on various levels of language granularity, the alignment can be conducted at the word, phrase, and sentence level^6^. Sentence alignment is considered a fundamental task in multilingual text processing, which has resulted in the proposal of many sentence alignment algorithms. They can be classified into three approaches: length-based, lexicon-based, and translation-based approaches to sentence alignment^7–10^. The first two approaches rely on the assumption that the order and length of the sentences are relatively the same in bitext (aligned source and its corresponding target text). Both approaches have pros and cons, which motivated researchers (e.g. Refs.^9,11^) to combine length-based features with lexical similarities for aligning sentences^12,13^. Algorithms that are unsupervised and language independent use mainly sentence length statistics^14^. The length-based algorithms can achieve small error rates on literal translations, but in translations where some sentences are skipped or merged they are not robust^7^. Sennrich and Volk^5^ have presented an algorithm that can compute sentence alignment for a parallel text. This algorithm requires two sets of documents, which can be articles or paragraphs, along with their corresponding translations. The texts are separated by hard delimiters, and it is essential to choose reliable delimiters since the algorithm does not look for alignments that cross these boundaries. The algorithm is primarily designed for articles that cover multiple pages, usually containing 500 or more sentences. Although it is fast enough to process such long texts, its quadratic complexity makes it unsuitable for aligning entire text collections without the use of hard delimiters.

Sentence-aligned bitext is used to train nearly all machine translation (MT) systems. Alignment errors have been noted as having a small effect on statistical MT performance^15^. However, misaligned sentences have been shown to be much more detrimental to neural machine translation (NMT)^16^.

Despite the recent success of neural machine translation (MT) models with large amounts of data, Kim et al.^17^ demonstrate that both supervised and semi-supervised approaches outperform even the best unsupervised systems. Accurate alignment between parallel corpora is essential not only for machine translation but also for lexicography, terminology extraction, and other applications. In Digital Humanities (DH), parallel corpus alignment is used for historical language learning, version alignment of medieval texts, and more.

Due to the importance of alignment tasks in natural language processing (NLP), several sentence alignment tools have been developed^1^. Some aligners are more general and language independent, while other aligners are specific, (in certain domain such as noisy data) and language dependent (work between given languages)^11^. Seničić and Fairon^18^ divide the sentence alignment tools into three categories: (1) statistical aligners, (2) lexical aligners, and (3) hybrid aligners. Statistical aligners use length-based algorithms (e.g. Gale-Church algorithm) to determine equivalency between the sentences in the source and target language. They have been used in the alignment of the Europarl corpus. Lexical aligners use dictionaries or lexicons (lexical information) to determine equivalency between sentences in the source and target language (e.g. Champollion). Hybrid aligner combines statistical methods with available lexical information^8^. Hunalign performed very well for languages with high degrees of inflection such as Slovak or Hungarian.

Traditional sentence alignment approaches suffer from a sparsity problem due to language ambiguity^13^.

As neural networks have been shown to be a powerful approach to text representation and sentence modelling, automatic sentence alignment methods using neural networks have started to gain popularity^19–22^. Neural alignment maps a source sentence into a fixed-length vector and then predicts if two sentences are aligned by their sentence vectors^13^. Li^23^ classifies neural alignment approaches into induction, unsupervised, and guided approaches. The induction approach operates on the attentional NMT models^19^. The unsupervised approach does not train the alignment on gold sentence/word alignment^24^ and the guided neural approach to alignment utilizes training data with word/sentence alignments^17^.

### Related work

The Slovak language, which is the subject of our research, belongs to the Slavic language group. Specifically, it is classified as a West Slavic language, along with Polish, Czech, and partly Sorbian (Lusatian Sorbian). To our knowledge, there has not been significant attention given to sentence alignment in the context of the Slovak language compared to other Slavic languages. For example, Bojar and Prokopova^25^ assessed the accuracy of the GIZA +  + alignment toolkit for the Czech-English language pair. Kruijff-Korbayová et al.^26^ compared automatic (GIZA + +) and manual word alignment.

Similarly, Marecek et al.^27^ focused on the alignment of Czech and English tectogrammatical dependency trees, comparing the t-aligner with GIZA +  + . Ngo Ho^28^ analyzed two statistical word alignment systems, GIZA +  + and Fastalign, for six language pairs—English with French, German, Romanian, Czech, Japanese, and Vietnamese.

In recent times, there have been several research efforts focusing on low resource languages, such as Slavic languages. These studies have aimed to propose new alignment methods, such as using deep learning networks for bilingual sentence alignment based on sentence embeddings such as Vecalign^2^ or multilingual sentence embeddings^29^, pre-trained multilingual language models based on the BERT architecture^30^, or machine translation^31^.

Tien et al.^32^ evaluated four alignment methods (Champolion, Hunalign, Vecalign, and a combination of Vecalign and Laser) for the Vietnamese-Lao language pair. Fernando et al.^33^ conducted an evaluation of sentence representations derived from LASER, XLM-R, and LaBSE with Hunalign on three language pairs: Sinhala–Tamil, Sinhala–English, and Tamil–English. Signoroni and Rychlý^34^ evaluated four alignment methods (Gale&Church, Hunalign, Bleualign, and Vecalign) for the English-Yorùbá language pair. A. K. Singh and Husain^35^ evaluated four alignment methods (Brown, Gale&Church, Melamed, and Moore) for the English-Hindi language pair. Abdul-Rauf et al.^36^ evaluated five different alignment methods (Gale&Church, MBA, Hunalign, Bleualign, and Gargantua) on French–English and Urdu-English bitexts. Krynicki^37^ assessed the performance of four sentence aligners (Moore, Hunalign, Bleualign, and Gargantua) on English-Polish bitexts.

Graen^38^ conducted research comparing the alignment of sentences in multiparallel corpora, primarily based on the Europarl corpus, using a multilingual sentence alignment algorithm. The performance of this algorithm was compared with the Hunalign algorithm. However, the set of gold alignments only included six languages, and Slovak was not among them. The evaluation considered not only the coverage of multilingual sentence alignment, but also compared these alignments using the F-score.

In this paper, we compare different alignment approaches and present our custom algorithm for alignment evaluation against human-aligned reference, which is a typical approach for evaluation^12^. In the next section we offer a brief overview of the different sentence alignment tools. The third section describes a methodology and the extra steps and tools that were required in order to create a unified output from different aligners. In the fourth section we present the results with the last section summarizing the findings.

## Sentence alignment

Early sentence alignments^7,8^ use scoring functions based only on the number of words or characters in each sentence and alignment algorithms based on dynamic programming (DP). DP is *O* time complexity ($O=N\times M$), where *N* and *M* are the number of sentences in the source and target documents.

The state-of-the-art aligners share a two-step algorithm (in decoding). The first focuses on extraction of parallel sentences, which the system considers reliable. These extracted parallel sentences are then used as anchor points to reduce search space or to obtain better estimation tools for parallelism (unsupervised) or both. The second step relies on realignment using the information obtained from the first step. Both algorithms arise from the following assumptions: only a limited number of alignment link types exist, and these links lie around the diagonal^39^.

Some sentence alignment models are supervised^22^, depend on dictionaries or existing sentence pairs and are weak in many to many sentence alignment.

### Bilingual sentence aligner

Moore^9^ presents a three-step algorithm that blends techniques adapted from previous work on word and sentence alignment. Initially, the algorithm aligns the corpus by utilizing a modified version of Brown et al.’s^40^ sentence-length-based model. Next, Moore^9^ incorporates an innovative search-pruning method to efficiently identify the most probable sentence pairs without relying on anchor points or previously aligned units. He then employs the sentence pairs assigned the highest probability of alignment to train a revised edition of IBM Translation Model 1. Lastly, he realigns the corpus, augmenting the initial alignment model with IBM Model 1, to generate an alignment that is based on both sentence length and word correspondence. The search is restricted to the minimal alignment segments that were assigned a non-negligible probability based on the initial alignment model. As a result, the search space is reduced significantly, making this final alignment quicker than the initial alignment, despite the model being more computationally demanding for each segment. This method employs both sentence length and lexical correspondence to derive the final alignment. However, since the lexical correspondence is obtained automatically, no externally supplied lexicon is required.

### Hunalign

Hunalign is a tool that aligns text in two languages on a sentence level. It requires tokenized and sentence-segmented text as input and produces bilingual sentence pairs (bisentences) as output. In case a dictionary^10^ is available, Hunalign utilizes it to combine with Gale-Church sentence-length information. However, if a dictionary is not available, it falls back to sentence-length information and creates an automatic dictionary based on the alignment. In the second pass, Hunalign uses the automatic dictionary to realign the text. It’s important to note that, similar to other sentence aligners, Hunalign cannot handle changes in sentence order and cannot generate crossing alignments where segments A and B in one language correspond to segments B’ and A’ in the other language.

Moore’s aligner^9^ and Varga et al.’s^11^ approach both employ a two-pass algorithm for aligning bilingual text, where a length-based method is used in the initial alignment. The first alignment is then utilized as training data for a translation model, which is subsequently applied to a complex similarity score. The main difference between these two approaches lies in the type of translation model used. Varga et al.’s approach utilizes a dictionary-based translation model, which can be manually expanded^20^, while Moore’s aligner^9^ works with the IBM-1 translation model.

### Bleualign

The alignment process involves calculating a similarity score and is completed in two steps. In the first step, the algorithm searches for 1-to-1 alignments to maximize the BLEU score. The remaining sentences are then aligned using a 1-to-n approach or a length-based algorithm^5^.

Bleualign utilizes machine translations and the BLEU score as a similarity metric to locate reliable anchor points. The gaps between these points are then filled in using BLEU-based and length-based heuristics. According to the authors^5^, this method surpasses state-of-the-art algorithms in alignment tasks and produces better performance in statistical machine translation (SMT). The Bleualign algorithm is executed for every text segment between two hard delimiters (including the beginning and end of the file) and comprises of two stages. In the first stage, a group of anchor points is identified using the BLEU score between the translated source text and the target text. The second step involves either aligning the sentences between these anchor points using BLEU-based heuristics or applying the length-based algorithm developed by Gale and Church^21^.

### Vecalign

Vecalign is a quick and precise method for aligning sentences, even for lengthy documents. When used alongside LASER, it can be applied to over 100 languages (i.e. 100^2 language pairs) without relying on a machine translation system or lexicon.

Vecalign employs multilingual sentence embeddings to measure the similarity between sentences and uses an approximation of Dynamic Programming (DP) based on Fast Dynamic Time Warping, which has a linear time and space complexity in relation to the number of sentences being aligned^2^. This allows Vecalign to efficiently align long documents in multiple languages, without relying on a machine translation system or lexicon.

Vecalign introduces a novel scoring function that measures the similarity of bilingual sentence embeddings. This approach calculates scores for the similarity of sentence embeddings by employing cosine similarity and normalizing it with randomly selected embeddings. It then averages neighboring pairs of sentence embeddings in both documents and aligns these approximate embeddings. This alignment is subsequently fine-tuned iteratively, using the original embeddings and a small window around them^23^.

The LASER4 tool is used to compute the sentence embeddings, which are based on an architecture for creating language-independent sentence embeddings^41^.

Thompson and Koehn^2^ evaluated sentence alignment accuracy using the development/test split released with Bleualign, consisting of manually aligned yearbook articles published in both German and French by the Swiss Alpine Club from the Text + Berg corpus^5^. Hyperparameters were chosen to optimize F1 on the development set. They considered alignments of up to 6 total sentences, which means they allowed alignments of size $Q-R$ where $Q+R\le 6$. The authors compared their approach to Refs.^5,8,9^, and the Coverage-based sentence alignment tool^42^. Hunalign was used in both bootstrapping mode as well as using a publicly available De–Fr lexicon from OPUS created from Europarl.

### Bertalign

Bertalign^43^ is a novel solution aimed at improving the accuracy of sentence alignment, particularly focusing on literary texts. Bertalign introduces a two-step algorithm for bilingual sentence alignment. In the first step, it identifies optimal one-to-one alignments by leveraging bidirectional encoder representations from transformer-based cross-lingual word embeddings. Specifically, it selects the top-k most semantically similar target sentences for each source sentence. In the second step, the Bertalign utilizes the paths found in the first step to recover all valid alignments that involve more than one sentence on each side of the bilingual text. The Bertalign aligner supports 25 languages, including the Slovak language.

As the Liu and Zhu^43^ state, literary texts pose unique challenges in the alignment process as they often involve complex, interpretative translations that do not neatly correspond to one-to-one mappings between source and target sentences. Traditional alignment methods tend to emphasize one-to-one links, making it challenging to handle the more intricate one-to-many and many-to-many alignments prevalent in literary content.

Liu and Zhu^43^ compared Bertalign’s results with five baseline systems, including Gale-Church, Hunalign, Bleualign, Bleurtalign, and Vecalign. They demonstrate that Bertalign achieves the highest accuracy, measured by the F1 score, on both evaluation datasets compared to up to now approaches and methods.

## Methods

The goal of the research is to evaluate the performance (Total alignments, Matched alignments, Precision (1), Recall (2), and F1 score (3)) of different alignment algorithms (BSA, Hunalign, Bleualign, Vecalign, and Bertalign) for the Slovak-English language pair. To achieve this goal, we required a dataset (corpus) that was not pre-aligned. Consequently, we opted not to utilize any pre-existing parallel corpora (or aligned multilingual or bilingual datasets), as they are already aligned. We carried out an experiment on 50 documents (dataset) while investigating the performance of individual algorithms. The performance of each algorithm (tool) was measured using the precision, recall and f-score metrics. Our dataset has 50 cases and 25 variables (5 performance measures × 5 algorithms).

We created our custom dataset (corpus), which consists of texts collected in 2021 and 2022. The obtained texts contain English texts of various genres and their human translations into Slovak. In total, we obtained 86 English documents (10,814 sentences) and 86 corresponding, but not aligned Slovak translations (12,220 sentences); in this study we use only 50 of them (50 originals and 50 corresponding not aligned translations). We manually aligned all the examined texts to obtain a reference sentence alignment, which we later used for computing the performance measures. Manual sentence alignment was conducted by three professional translators. To guarantee the quality of the human-aligned reference, we conducted a thorough review of the outcomes generated by the tool designed for assessing aligners. Any disparities detected by the tool between the automatically aligned content and the human-aligned reference were manually inspected and corrected within the reference alignment in cases where errors were identified.

We state the global null statistical hypotheses for the performance measures:

### H0: 

Accuracy of the alignment (Total alignments/Matched alignments/Precision/Recall/F1 score) does not depend on the used alignment algorithm/tool (BSA, Hunalign, Bleualign, Vecalign, or Bertalign).

The number of total alignments is the number of alignments created by the tool and the number of matched alignments is the number of alignments that were found in both reference alignments and in alignments created by the tool.

1 $$\mathrm{Precision }= \frac{\mathrm{matched\, alignments}}{\mathrm{total\, number\, of \,alignments}}$$

2 $$\mathrm{Recall }= \frac{\mathrm{matched \,alignments}}{\mathrm{reference\, alignments}}$$

3 $$\mathrm{F}1\;\mathrm{ Score }= 2*\frac{\mathrm{Precision }*\mathrm{ Recall}}{\mathrm{Precision }+\mathrm{ Recall}}.$$

We have created our own tool that uses a simple algorithm to evaluate sentence alignments. It takes the alignment created by the aligner for source text, reads the first line and then searches in reference for source text (manually aligned). If a match is found, it takes the target alignment from reference and searches for a match in the target alignment file created by the aligner. If no match is found in the source texts, it is evaluated as a wrong alignment, since the reference alignment for the source text does not contain the automatic alignment created by the aligner.

### Tools: BSA, Hunalign, Bleualign, Vecalign, and Bertalign 

We took the latest version available on github and used local builds (in case of Hunalign). The only exception was BSA from Microsoft, which is not open-source and we used download link on their official webpage. In most of the cases we have used default parameters and configurations. For Hunalign, we did use the dictionaries collected from https://github.com/coezbek/hunalign-dict-muse. We utilized the code from https://github.com/rsennrich/Bleualign for sentence alignment with Bleualign, and https://github.com/thompsonb/vecaalign for sentence alignment with Vecalign. Additionally, we used https://github.com/bfsujason/bertalign for sentence alignment with Bertalign.

**Figure 1:**
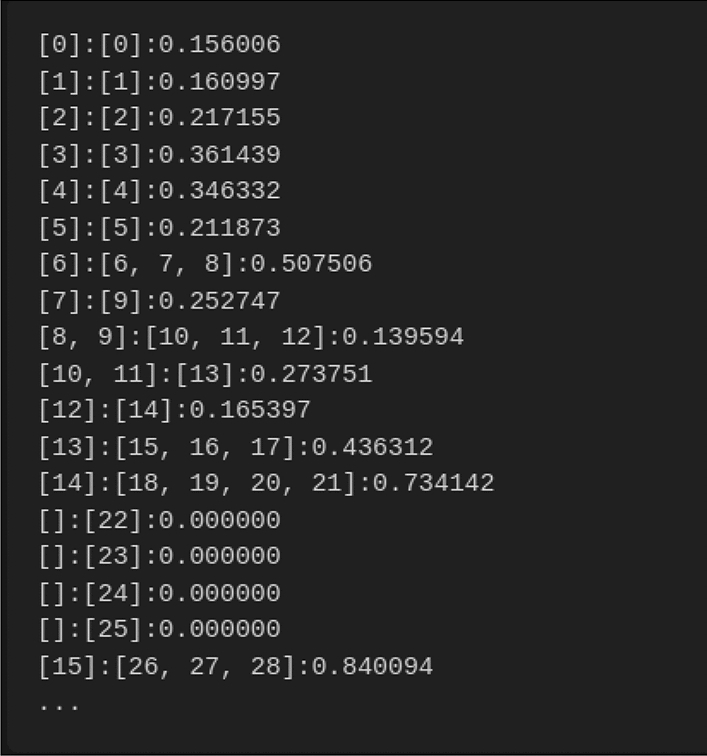
Vecalign’s output format.

**Figure 2:**
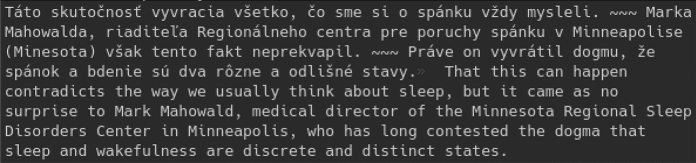
Example of Hunalign output.

Different tools use different output formats. Some of them return aligned text files (e.g. Bleualigner), some return a table with aligned sentence indexes (e.g. Vecalign, Fig. 1) and others produce a single file where alignments are separated by delimiters (e.g. Hunalign, Fig. 2).

Since our human aligned reference files are two plain text files and our evaluation algorithm requires the alignment results to be in two separate plain text files, we had to post-process some of the immediate results from alignment tools. Of the tested tools, only Bleualigner, BSA, and Bertalign do not require any post-processing effort. They return two sentence aligned plain text files as their output.

### Vecalign post-processing

As shown, the sentence alignment output from Vecalign includes the source and target sentence indexes for each alignment, and a sentence alignment cost which incorporates normalization but not penalties for containing multiple sentences.

To create a more meaningful output, we created an algorithm (https://github.com/4gac/aligner_eval), that can process the result from Vecalign and create a parallel corpus based on Vecalign results. Based on the information from the first entry and the source file, we can create a new text document, in which the sentences in the lines are written according to the instructions from Vecalign. Then, we repeat the algorithm for the second entry in the vecalign output and target file. As a result, we obtain a pair of sentence aligned text files.

### Hunalign post-processing

Hungalign creates a single text file containing the aligned source and target sentences separated by a tab (\t). It also contains triple tilde (~ ~ ~) in cases where the alignment between source and target was not 1:1 (Fig. 2).

We wrote a simple program to post-process this output. We had to remove triple tildes, because the evaluation process would not be possible as the strings in output files would be different than those in the original files. Following this we created two aligned text files to match the specified output format.

## Results

The aim of the research was to assess the performance (Total alignments, Matched alignments, Precision, Recall, F1 score) of different alignment algorithms used in tools (BSA, Bleualign, Vecalign, Hunalign, and Bertalign) for the language pair Slovak-English. The experiment was conducted on 50 documents, with a focus on evaluating the performance of each algorithm. In other words, our dataset consists of 50 cases and 25 variables (5 performance measures × 5 algorithms).

We established global null statistical hypotheses for the observed performance measures:

### H0: 

The correctness of alignment (Total alignments/Matched alignments/Precision/Recall/F1 score) does not depend on the alignment algorithm used (BSA, Bleualign, Vecalign, Hunalign, Bertalign).

Given the small deviations from normality, the sufficient number of cases and the robustness of the F test statistic against the violation of the assumption of normality, we decided to use parametric procedures. To test the global null hypotheses, in the case of Total alignments, Matched alignments, Precision, Recall, and F1 score, we used adjusted univariate tests for repeated measures (Huynh–Feldt correction) due to violation of the sphericity assumption (Total alignments: *W* = 0.001, *Chi-sqr* = 338.714, *df* = 9, *p* < 0.001, Matched alignments: *W* = 0.042, *Chi-sqr* = 150.843, *df* = 9, *p* < 0.001, Precision: *W* = 0.132, *Chi-sqr* = 96.130, *df* = 9, *p* < 0.001, Recall: *W* = 0.539, *Chi-sqr* = 29.271, *df* = 9, *p* < 0.001, F1 score: *W* = 0.516, *Chi-sqr* = 31.395, *df* = 9, *p* < 0.001), F1 score: *W* = 0.769, *Chi-sqr* = 12.506, df = 5, p < 0.05).

In the case of Total alignments, Matched alignments, Precision, Recall, and F1 score, we reject the null hypothesis at the 0.001 significance level, based on the results of adjusted univariate tests for repeated measures (Total alignments: *H-F Epsilon* < 0.29, *H-F p* < 0.001, Matched alignments: *H-F Epsilon* < 0.63, *H-F p* < 0.001, Precision: *H-F Epsilon* < 0.60, *H-F p* < 0.001, Recall: *H-F Epsilon* < 0.82, *H-F p* < 0.001, F1 score: *H-F Epsilon* < 0.80, *H-F p* < 0.001).

After rejecting the global null hypotheses, we identified homogeneous groups and statistically significant differences in alignment accuracy (Total alignments/Matched alignments/Precision/Recall/F1 score) between the examined algorithms (BSA, Hunalign, Bleualign, Vecalign, and Bertalign). For this purpose, we used multiple comparisons, specifically the Duncan’s test, which is more appropriate compared to standard post-hoc tests.

From the point of view of the total number of alignments (Table 1a), the Hunalign tool performs statistically significantly the best (*p* < 0.05) and the BSA tool performs the worst (*p* < 0.05). The Bertalign, Vecalign, and Bleualign tools, in terms of the total number of alignments (Table 1a) form a one homogeneous group (*p* > 0.05). However, if we look at the number of matched alignments (Table 1b), the Bertalign tool performed best. A statistically significant difference in favour of the Bertalign tool (Table 1b) was shown against the Bleualign and BSA tools (*p* < 0.05), on the contrary, a statistically significant difference was not proved between the Bertalign, Vecalign, and Hunalign tools (*p* > 0.05), these tools formed a one homogeneous group in terms of the number of matched alignments. The BSA achieved the lowest number of alignments in both cases (Table 1a,b).

**Table 1:** Multiple comparisons: (a) Total alignments, (b) Matched alignments.

Total alignments	Mean	StdDev	1	2	3	Matched alignments	Mean	StdDev	1	2	3
BSA	188.06	64.24		***		BSA	166.92	61.79			***
Bleualign	212.12	72.40	***			Bleualign	187.38	76.15		***	
Bertalign	215.46	74.85	***			Hunalign	199.78	67.56	***	***	
Vecalign	215.76	73.82	***			Vecalign	201.74	67.21	***		
Hunalign	220.14	76.14			***	Bertalign	209.74	71.54	***		

***Homogeneous group (*p* > 0.05).

In terms of the performance of accuracy measured by Precision and Recall, the results are similar (Table 2a,b). In terms of Precision (Table 2a), the Hunalign, Bleualign, and BSA algorithms achieved the same performance (*p* > 0.05) and the best performance was achieved by the Bertalign tool, which is statistically significantly better than the Hunalign, Bleualign, and BSA tools (*p* < 0.05). On the other hand, a statistically significant difference was not demonstrated between the Bertalign and Vecalign algorithms (p > 0.05); these tools form a one homogeneous group in terms of the performance of accuracy measured by Precision. Similar results were also proved in the case of Recall (Table 2b), where the best performance was achieved by the tools Bertalign and Vecalign. Both form a one homogeneous group in terms of performance of accuracy in dependency on reference sentence alignment (*p* > 0.05).

**Table 2:** Multiple comparisons: (a) Precision, (b) Recall.

F1 score	Mean	StdDev	1	2	3	Recall	Mean	StdDev	1	2	3	4
BSA	0.88	0.12	***			BSA	0.78	0.14				***
Bleualign	0.89	0.19	***			Bleualign	0.88	0.19	***			
Hunalign	0.91	0.06	***	***		Hunalign	0.91	0.13	***	***		
Vecalign	0.95	0.12		***	***	Vecalign	0.95	0.12		***	***	
Bertalign	0.98	0.02			***	Bertalign	0.97	0.02			***	

***Homogeneous group (*p* > 0.05).

However, if we look at the performance of the tools in terms of the harmonic mean of the Precision and Recall, i.e. F1 score (Table 3), the Bertalign and Vecalign tools perform statistically significantly best (*p* < 0.05) and the BSA tool performs the worst (*p* < 0.05). The Hunalign and Bleualign tools achieved the same performance in terms of F1 score and form a one homogeneous group (*p* > 0.05).

**Table 3:** Multiple comparisons—F1 score.

F1 score	Mean	StdDev	1	2	3
BSA	0.82	0.13			***
Bleualign	0.88	0.19	***		
Hunalign	0.90	0.13	***		
Vecalign	0.95	0.12		***	
Bertalign	0.97	0.02		***	

****Homogeneous group (*p* > 0.05).

The highest variability (Tables 1b, 2, and 3), based on the performance measured by Match alignments, Precision, Recall, and F1 score, was identified for the Bleualign tool. On the other hand, the Bertalign tool achieved the lowest variability in performance measured by Precision, Recall, and F1 score (Tables 2 and 3). The Bleualign achieved the most diverse results in terms of performance of accuracy depending on reference sentence alignment.

**Figure 3:**
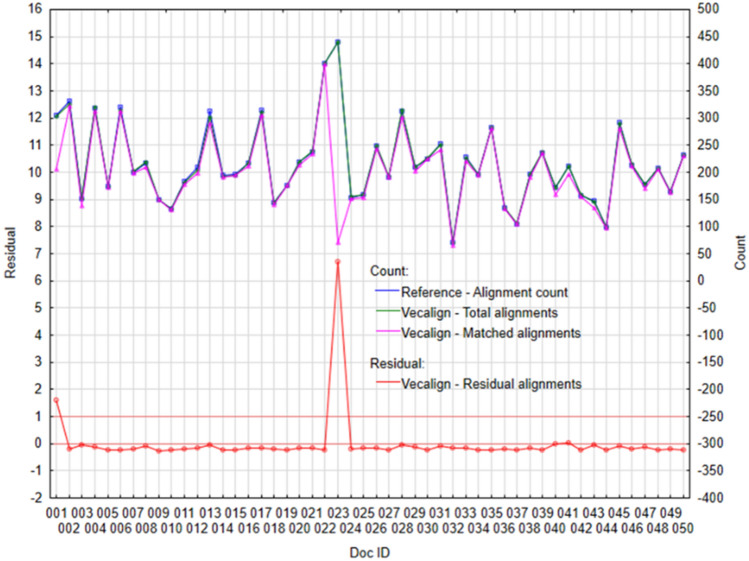
Plot of selected variables for algorithm Vecalign: (Left) Standardized residual alignments, (Right) Total alignments, Matched alignments, and Reference—alignment count.

**Figure 4:**
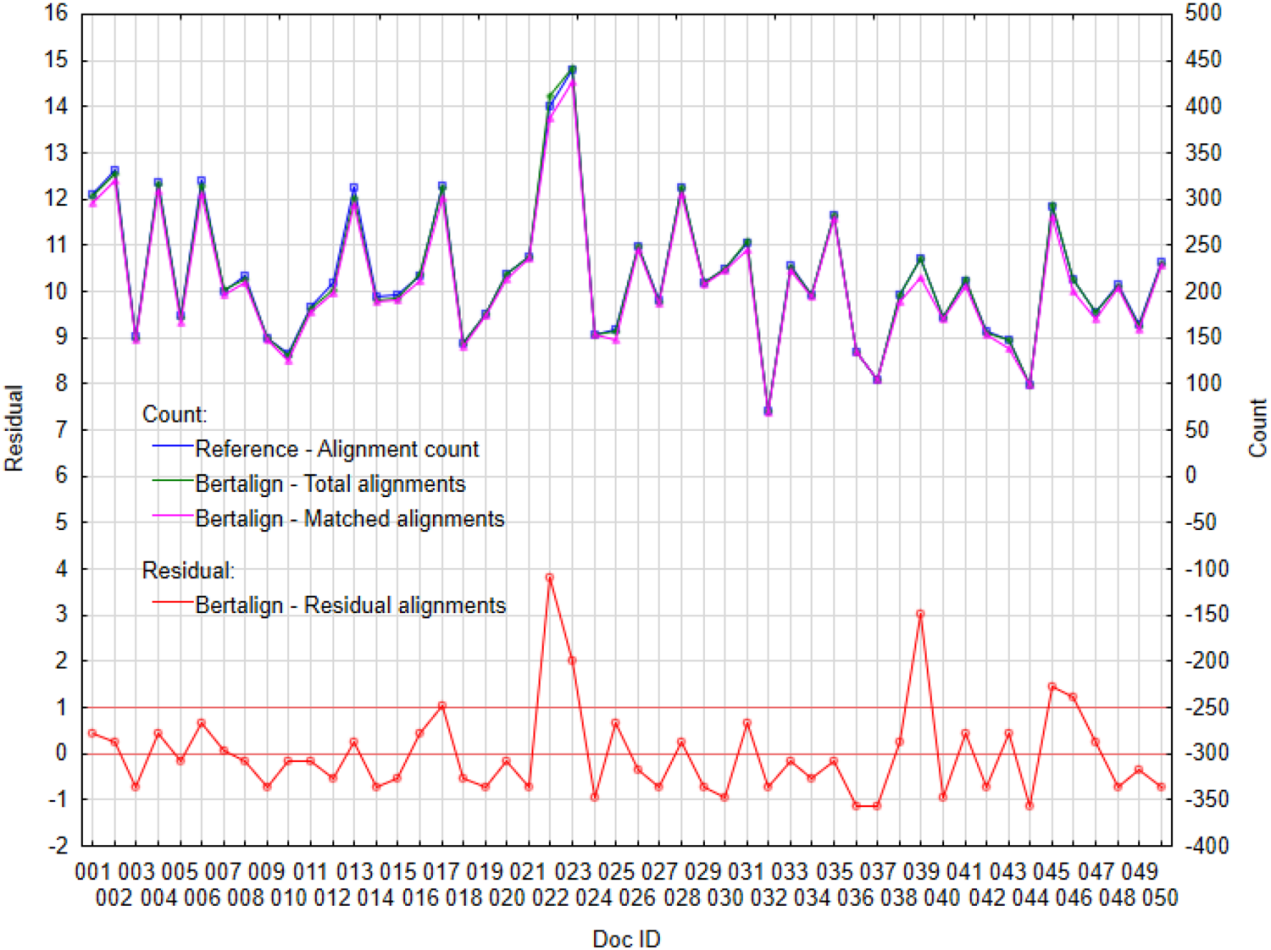
Plot of selected variables for algorithm Bertalign: *(*Left) Standardized residual alignments, *(*Right) Total alignments, Matched alignments and Reference—alignment count.

**Figure 5:**
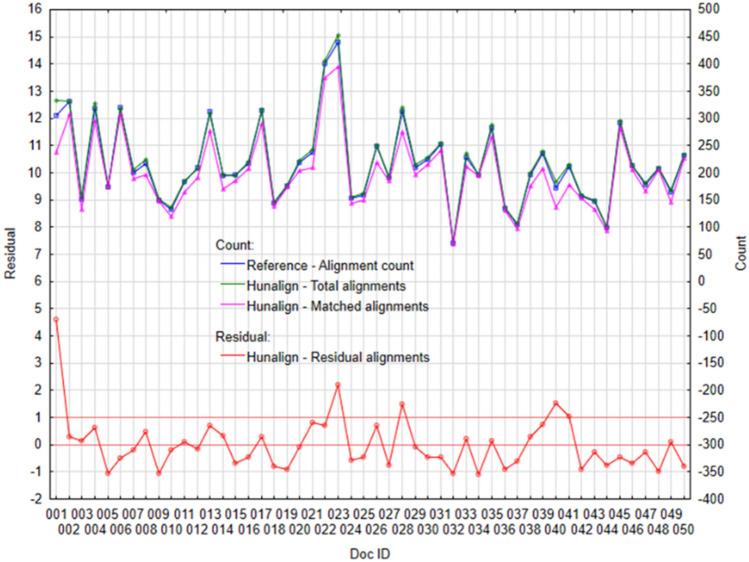
Plot of selected variables for Hunalign: (Left) Standardized residual alignments, (Right) Total alignments, Matched alignments and Reference—alignment count.

**Figure 6:**
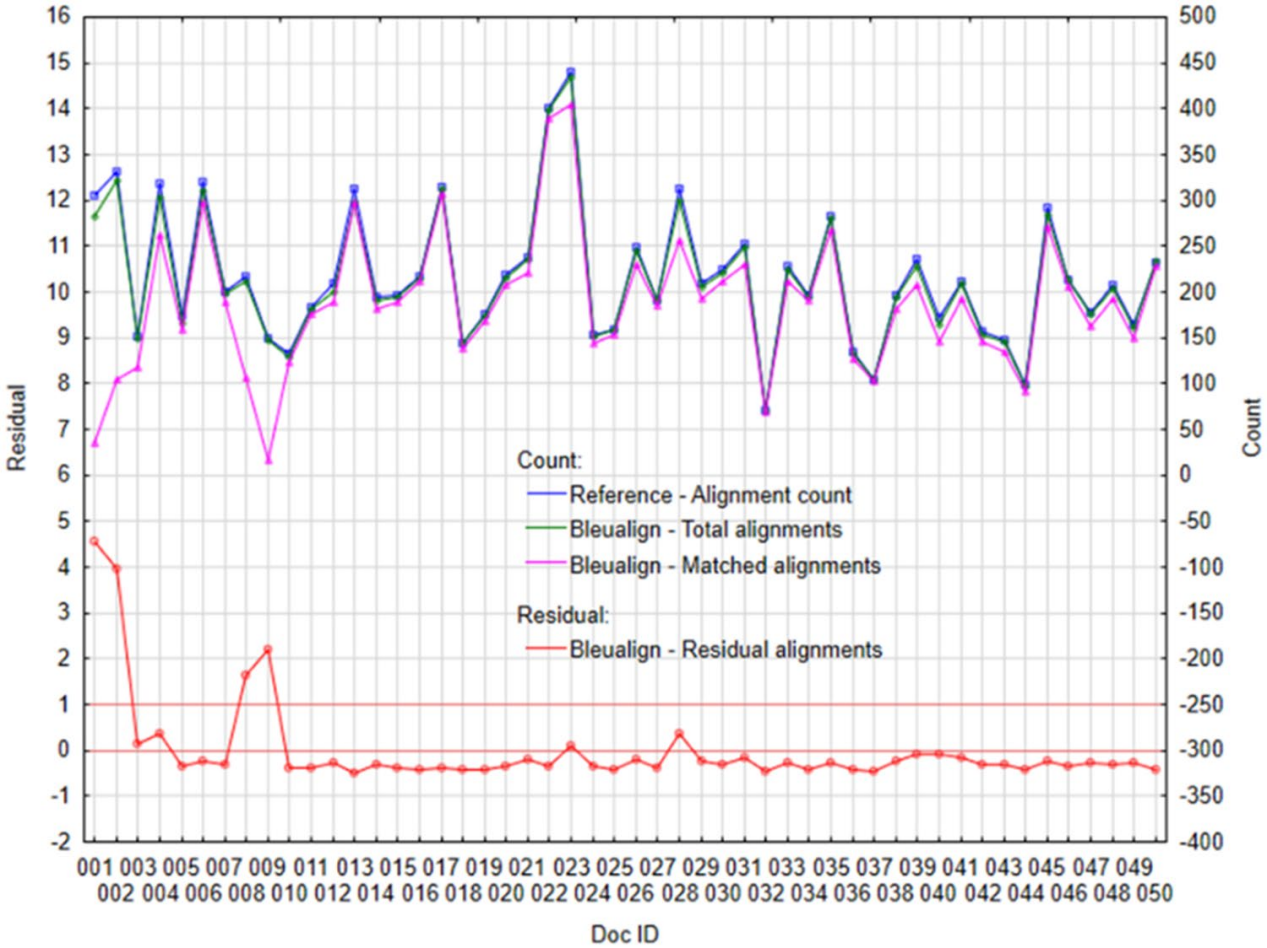
Plot of selected variables for Bleualign: (Left) Standardized residual alignments, (Right) Total alignments, Matched alignments and Reference—alignment count.

If we look at the performance of individual tools in more detail, through individual cases—documents. The following figures (Figs. 3, 4, 5 and 6) visualize the Residual alignments (left axis), where the residuals represent the difference between Total alignments and Matched alignments, which we subsequently standardized with the mean and standard deviation. On the right axis (Figs. 3, 4, 5 and 6) there are values for individual counts (Reference—alignment count, Total alignments, and Matched alignments).

These results confirm the previous findings; the best performance is achieved by the Vecalign (Fig. 3) and Bertalign (Fig. 4). Individual counts (Figs. 3, 4) are copied (Reference—alignment count, Total alignments and Matched alignments). In the case of Vecalign (Fig. 3), we identified larger differences for two documents, ID#001 and ID#023 (residual > 1), while for Bertalign (Fig. 4) five documents, ID#022, ID#023, ID#039, ID#045, and ID#046, showed differences greater than 1.

In document ID#023 Vecalign returned one of the alignments in the form []:[99]:0.000, which means that it aligned an empty English sentence to one Slovak sentence. After that all the alignments were wrongly aligned. In the original form, this document contains 440 English and 530 Slovak sentences respectively. A larger difference between the two may be the cause of such an error.

Similarly, document ID#001 contains 303 source sentences and 384 target sentences, which is a difference of 81 sentences. In this case we can also see a higher number of residual alignments, which further supports our assumption.

This could be fixed by splitting the document into smaller batches (paragraphs).

Based on performance measures (Matched alignments, Precision, Recall, and F1 score), the Hunalign and Bleualign achieved approximately the same performance, which was also confirmed by a more detailed look at the results, through individual documents (Figs. 5, 6). In both cases (Figs. 5, 6) the individual counts are copied (Reference—alignment count, Total alignments, and Matched alignments), although with higher deviations as compared to Vecalign. In the case of Hunalign (Fig. 5), we identified greater differences in the case of four documents ID#001, ID#023, ID#028, and ID#040 (*residual* > 1). Similarly, in the case of Bleualign (Fig. 6), we identified greater differences also in the case of four documents ID#001, ID#002, ID#008, and ID#009 (*residual* > 1).

We identified a few cases where the Bleualign tool misaligned sentences early in the process and was unable to recover. The rest of the sentences were misaligned accordingly and that might be the cause of the higher standard deviation.

**Figure 7:**
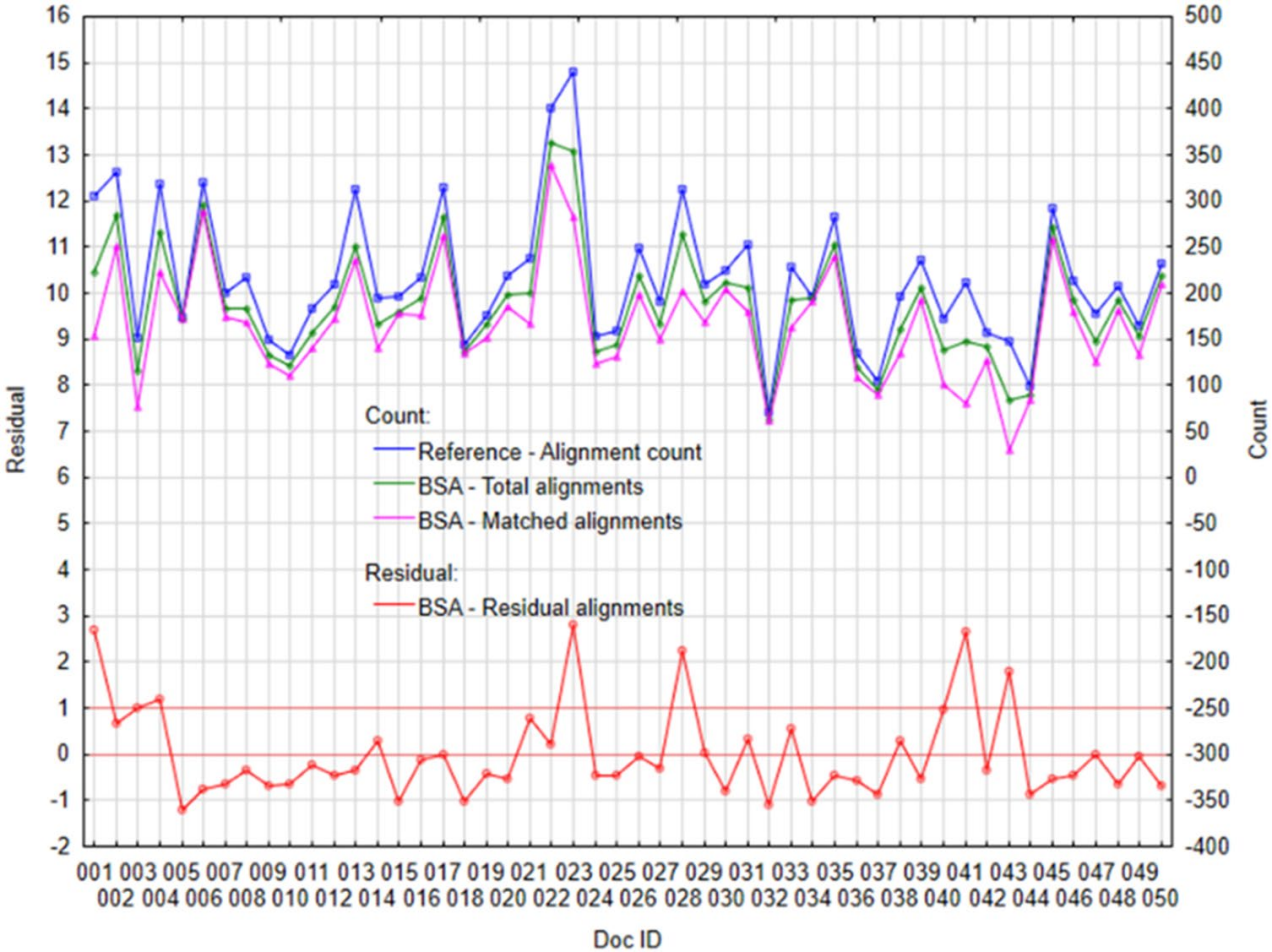
Plot of selected variables for BSA: (Left) Standardized residual alignments, (Right) Total alignments, Matched alignments and Reference—alignment count.

The worst performance is achieved by BSA (Fig. 7), although individual counts are copied (Reference—alignment count, Total alignments and Matched alignments), but significantly with the highest deviations. In the case of BSA (Fig. 7), we identified larger differences in the case of six documents ID#001, ID#004, ID#023, ID#028, ID#041, and ID#043 (*residual* > 1).

## Conclusion

This study provides an evaluation of five tools that are used to create automatic sentence alignments. We showed that both Bertalign and Vecalign, which requires only bilingual sentence embedding and are linear in time and space with respect to the number of sentences being aligned, outperform the previous state-of-the-art approaches and methods, which have a quadratic time complexity and requires a MT system.

Our results indicate that the currently designed Bertalign tool achieves the best performance in alignment accuracy measured by Precision, Recall, F-score, and Match alignment with respect to reference alignment. However, a statistically significant differences were proven only between the Bertalign and BSA/Hunalign/Bleualign. The Bertalign and Vecalign achieve approximately the same performance in alignment accuracy measured by Precision, Recall, F-score and, Match alignment with respect to reference alignment. In terms of Total alignment, the Bertalign did not achieve the best performance and there was no statistically significant difference between the Bertalign and Vecalign/Bleualign in the performance of the alignment accuracy with respect to the reference alignment.

We attempt to create a fair performance evaluation of sentence alignment tools. For this reason, we created multiple utility tools to obtain the results in the same output format.

We showed that the method compares the output against a human aligned reference based on strings, but it does not take into consideration that the reference is not always perfect. This should be fixed in the future, either by improving the algorithm or manually checking each reported misaligned sentence pair, which would be time consuming, since most of the misalignments are correctly reported.

According to Sennrich and Volk^5^, Bleualign’s performance is significantly influenced by the quality of translation provided. In the absence of any translation, where the algorithm calculates sentence similarity between the target and source texts directly, its performance is lower than Gale and Church on the same evaluation set. This is due to the limited number of sentence alignments identified by BLEU, with some of them being inaccurate. In some cases (ID#001, ID#002, and ID#009), the tool was unable to recover after making a wrong alignment at the beginning and therefore the rest of the sentences were also wrongly aligned, resulting in bad performance. Changing the MT systems might improve the result, but for our experiments we only used MT output as provided by Google Translate.

The main limitation we identified in automatic alignment is a decrease in alignment quality as the difference in the number of lines or sentences between the source and target increases. To address this limitation, we would suggest aligning smaller text chunks as a potential solution.

Another limitation of these tools is that almost all of them (except those that use length-based methods) depend on some other external tool. Bleualigner requires MT output, which must be generated by available tools and can be expensive with larger data sets. Hunalign requires a dictionary and Vecaling requires sentence embedding in a special.emb file format, which is generated by Facebook’s LASER^29^. Installing LASER itself consists of downloading encoders from Amazon s3, downloading external tools and then calculating vector embeddings for larger files which also takes some time.

Lastly, there is the human aspect. Manually aligned references contained a few wrongly aligned sentence pairs, which we only found during testing of the evaluation algorithm. The tool reports –f –verbose flag is enabled) possibly misaligned sentences in source or target reference. We manually checked for false reports from the tool, and we found incorrect alignment in the reference. After fixing the reference, the tool no longer reported misalignment and the measurements for aligners therefore improved.

## Data Availability

The datasets generated during and/or analyzed during the current study are available from the corresponding author on reasonable request.

## Abbreviations

MT: Machine translation
DH: Digital humanities
NLP: Natural language processing
NMT: Neural machine translation
DP: Dynamic programming
ILP: Integer linear programming
BSA: Bilingual sentence aligner
SMT: Statistical machine translation
